# Dendritic Cell Density and Morphology Can Be Used to Differentiate Vernal Keratoconjunctivitis from Allergic Conjunctivitis

**DOI:** 10.3390/biom13101469

**Published:** 2023-09-29

**Authors:** Zahra Tajbakhsh, Blanka Golebiowski, Fiona Stapleton, Ramin Salouti, M. Hosein Nowroozzadeh, Mohammad Zamani, Nancy Briggs, Isabelle Jalbert

**Affiliations:** 1 School of Optometry and Vision Science, UNSW, Sydney, NSW 2052, Australia; b.golebiowski@unsw.edu.au (B.G.); f.stapleton@unsw.edu.au (F.S.); i.jalbert@unsw.edu.au (I.J.); 2 Department of Optometry, School of Allied Health, University of Western Australia, Crawley, WA 6009, Australia; 3 Salouti Cornea Research Center, Salouti Eye Clinic, Shiraz 71839-33636, Iran; saloutir@hotmail.com (R.S.); amiragho@yahoo.com (M.Z.); 4 Department of Ophthalmology, School of Medicine, Shiraz University of Medical Sciences, Shiraz 71348-14336, Iran; norozzadeh@gmail.com; 5 Stats Central, Mark Wainwright Analytical Centre, UNSW, Sydney, NSW 2052, Australia; nancy.briggs@unsw.edu.au

**Keywords:** dendritic cell, allergic conjunctivitis, vernal keratoconjunctivitis, in vivo confocal microscopy, antigen capture capacity, migratory capacity

## Abstract

The aim of the study was to compare the distribution of corneal and conjunctival epithelial dendritic cells (DCs) in vernal keratoconjunctivitis (VKC), allergic conjunctivitis (AC), and non-allergic controls to examine if the allergy type causes differences in immune cell activation. The prospective study included 60 participants: 20 with VKC, 20 with AC, and 20 non-allergic controls. In vivo confocal microscopy was performed on the right eye. The locations scanned included the corneal centre, inferior whorl, corneal periphery, corneal limbus, and bulbar conjunctiva. The DCs were counted manually, and their morphology was assessed for the largest cell body size, the presence of dendrites, and the presence of long and thick dendrites. The DC density was higher in VKC and AC compared to non-allergic group at all locations (*p* ≤ 0.01) except at the inferior whorl. The DC density in VKC participants was significantly higher than in AC at the limbus (*p* < 0.001) but not at other locations. Both the AC and the VKC group had larger DC bodies at the corneal periphery and limbus compared to the non-allergic group (*p* ≤ 0.03). The study found a higher proportion of participants with DCs exhibiting long dendrites at both the corneal periphery in AC (*p* = 0.01) and at the corneal centre, periphery, and limbus in VKC, compared to the non-allergic group (*p* ≤ 0.001). In conclusion, a higher DC density at the limbus may be a marker of more severe VKC. DCs with larger cell bodies and a greater proportion of participants with DCs displaying long dendrites can be potential markers to differentiate allergy from non-allergy, and more severe forms of allergy from milder forms.

## 1. Introduction

Ocular allergy is one of the common disorders of the external ocular adnexa. Seasonal and perennial allergic conjunctivitis (AC) are prevalent, and relatively benign, forms of ocular allergic disease. Rarer, but typically more severe, forms of ocular allergic disease include atopic keratoconjunctivitis and vernal keratoconjunctivitis (VKC). Although seasonal and perennial AC are relatively milder, they account for the majority of ocular allergy cases (95% in the United States), and their resultant symptoms and signs carry a profound societal and economic burden [1,2]. Atopic keratoconjunctivitis and VKC are less common but typically more severe in presentation. A high prevalence of these sight-threatening diseases is found in warm and subtropical climates, including the Mediterranean, the Middle East, and Asian countries such as Japan [3,4,5].

Dendritic cells (DCs) are antigen-presenting cells that play an essential role in the initiation of allergic disease [6,7,8]. The seasonal and perennial forms of AC have an IgE-mediated mechanism, whereas VKC and atopic keratoconjunctivitis have more complex immunopathologic mechanisms that include both IgE- and non-IgE-mediated pathways [9]. In a mouse model of AC, a marked influx of conjunctival DCs has been reported following an allergen challenge. This suggests a potential role for conjunctival DCs in the immune response in AC [10]. The immunohistochemical analysis of human conjunctival biopsies has shown that CD4+ T helper type 2 cells activated by DCs play a key role in the pathogenesis of chronic and severe forms of VKC [4].

Confocal microscopy enables the observation of DCs in vivo at the ocular surface. Recent work by our group using in vivo confocal microscopy (IVCM) has shown an increased DC density at corneal and conjunctival locations, with an increased antigen capture capacity in the DCs, indicated by larger cell bodies and a higher proportion of long dendrites, in those with AC [11]. Studies that have assessed corneal and conjunctival DCs in VKC have previously reported an increased density of epithelial DCs in the central and far peripheral cornea, limbus, bulbar, and tarsal conjunctiva [12,13,14]. In addition, a higher DC density and DCs with longer dendrites and a larger cell field area (the size of the cell body and the length of the dendrites) at the corneal centre have been reported in quiescent VKC, compared to a non-allergic control group [15]. Similarly, our work has shown that the corneal DC density remains elevated during the asymptomatic phase of AC, but that the conjunctival DC density decreases compared to the active phase. However, these corneal DCs had smaller cell bodies with fewer dendrites during the asymptomatic phase of AC [16]. 

Recent studies have focused on DC morphology as an important indicator of DC activation, hypothesising that it may prove to be a more robust biomarker of disease than other clinical variables [17,18,19]. The differences in DC morphology between different types of ocular allergy have, to date, not been investigated. The differences in DC density between AC and the non-allergic control group was smaller than the differences between VKC and the control group reported in other studies [11,12,13,14]. The ocular surface DC density and morphology have, to date, not been directly compared in a single study that enrolled people with VKC and AC. 

Topographical variation in DC density and morphology exists at the normal ocular surface [20,21], and we have also shown topographical differences in immune activation in AC patients compared to control participants [11]. However, the topographical distribution of the DC density and morphology at various locations in the cornea and conjunctiva of VKC patients has not previously been investigated. 

Due to the differences in immunopathogenic mechanisms between VKC and AC, such a comparison may give insights into the differing underlying disease processes and immune responses between the two conditions. Such information ultimately could help to achieve better outcomes for the management of a wide spectrum of ocular allergies. 

This study, thus, aimed to assess and compare the distribution, density, and morphology of DCs at the cornea and conjunctiva of people with AC and VKC in order to identify the differences in immune activation in ocular allergies with different pathophysiological mechanisms.

## 2. Materials and Methods

### 2.1. Study Design and Participants 

A prospective, cross-sectional, observational study was conducted, involving 60 participants recruited from Salouti Eye Clinic, Shiraz, Iran. All the participants provided written informed consent, and the study adhered to the tenets of the Declaration of Helsinki. The Human Research Ethics Committee of UNSW Sydney (HC180930) and Shiraz University of Medical Sciences Ethics Committee (IR.SUMS.REC.1398.1247) approved the study. 

A convenience sample size of 20 participants in each of the three groups (VKC, AC, non-allergy) was considered. Participants who visited the clinic between March 2020 and October 2020 and who were diagnosed with AC or VKC were recruited for the study. Further, keeping in mind the ability of participants to follow instructions, only those aged 10 years or over were recruited. AC or VKC was diagnosed by attending ophthalmologists based on the published criteria [22,23]. Briefly, the criteria for a positive diagnosis were one or more characteristic symptoms (itchiness, redness, tearing), one or more characteristic signs (hyperaemia, conjunctival chemosis, conjunctival follicles (for AC and VKC), and the presence of Horner–Trantas dots (for VKC only)), and a history of seasonal exacerbations or a family history of ocular allergy. Non-allergic participants were recruited from patients who visited the eye clinic during the same period for a routine eye health check-up or for refractive surgery screening and who had healthy ocular structures. The non-allergic group was age-matched to the AC group only.

The exclusion criteria were the current use of topical or systemic anti-allergic medications comprising antihistamine/mast cell stabiliser/nasal corticosteroid sprays, the use of lubricants in the week before the study, current immunotherapy for aeroallergens, uncontrolled asthma, a past anaphylactic episode, regular wearing of contact lenses, any current ocular surface diseases other than ocular allergy, Sjøgren syndrome, active intraocular inflammation, a history of corneal refractive surgery/ocular surgery, current pregnancy or breastfeeding, and systemic conditions that can affect the ocular surface (for example, diabetes, thyroid disorder, or rheumatoid arthritis).

### 2.2. Ocular Surface Symptoms and Signs 

Participants’ symptoms were recorded using the Aston University Allergy Questionnaire (AUAQ) [24]. Limbal, bulbar, and palpebral redness were graded using the BHVI grading system [25]. Corneal epithelial disorders (superficial punctate keratitis/filamentary debris/shield ulcer), bulbar conjunctival chemosis, palpebral conjunctival papillae, and follicles were graded using the Japanese grading scale for allergic conjunctivitis [22].

### 2.3. In Vivo Assessment of DC Density and Morphology Using Confocal Microscopy

An HRT III confocal microscope with a Rostock Corneal Module (Heidelberg Engineering GmbH, Heidelberg, Germany) was used to capture images of corneal and conjunctival epithelial DCs in vivo from the right eye, as described earlier [20]. Five locations of the cornea and conjunctiva were scanned in the following order: corneal anatomical centre, corneal inferior whorl, far peripheral cornea (temporal, 1 mm inside limbus), limbal cornea (temporal), and bulbar conjunctiva (temporal, 2–3 mm from the limbus). The corneal sub-basal epithelium was detected at approximately 35–70 µm depth and the conjunctival epithelium at 5–20 µm depth. Prior to IVCM, the eye was anaesthetised using a topical anaesthetic (Anestocaine 0.5%, Sina Darou, Tehran, Iran). GenTeal gel (hypromellose 0.3%, carbomer 980 0.2%, Ciba Vision Ophthalmics, NSW, Australia) was used as a coupling medium between the objective lens and the Tomocap cap (Heidelberg Engineering, Heidelberg, Germany).

*Image analysis.* Five of the best-focused images, which overlapped by less than 20%, were selected from each corneal and conjunctival location; only one image from the inferior whorl was included, due to the small area. Bright hyperreflective cells of at least 10 µm in size, with a linear or curvilinear cell body, with or without dendrites (both short and long) located at the sub-basal corneal epithelium and distributed among nerve fibers, or in any layer of the conjunctival epithelium, were considered DCs [19]. The images were assessed for DC density and morphology by an experienced investigator masked to the participant group and the image location.

*DC density.* The DC density was counted manually, and the mean value of five images was recorded as cells/mm^2^, other than for the inferior whorl, where the value for a single image was recorded.

*DC morphology.* The DC morphology was graded using a validated grading system [19]. The cell body size was graded as small (10–25 µm), medium (26–40 µm), or large (>40 µm), based on the largest cell body size observed in any of the five images. The presence of any dendrites, the presence of long dendrites, and the presence of thick dendrites in any of the five images were recorded. In addition, the presentation of DCs in clusters with a wire netting pattern in the conjunctival epithelium was noted and recorded [13]. Images devoid of DCs were excluded from the morphology analysis.

### 2.4. Statistical Analysis

SPSS (version 26; SPSS Inc., Chicago, IL, USA) was used. The differences in symptoms and signs between the three groups were assessed using Kruskal–Wallis or ANOVA, as appropriate. The *p*-values of multiple comparisons between groups were adjusted using Holm’s step-down Bonferroni method.

The DC density was not normally distributed; therefore, the values were log-transformed after the addition of 0.2 to density values of zero. A linear mixed model with a random effect for individuals was used to examine differences in the DC density between groups and across corneal and conjunctival locations. The fixed effects for group and location and the interaction between group and location were included to examine the effect of location on the between-group differences in DC density. Pairwise comparisons between groups and between locations were obtained within the same model. The *p*-values of multiple comparisons between groups at each location and between locations for each group were adjusted using Holm’s step-down Bonferroni method. A pairwise comparison of the differences between locations was reported for the VKC group only.

Nonparametric analyses including the Kruskal–Wallis test (for cell body size), and Fisher’s exact test (for the presence of dendrites) were used to assess differences in DC morphology between groups. The Friedman and Wilcoxon signed-ranked test (for cell body size), and Cochran’s Q test (for the presence of dendrites) were used to assess the differences in DC morphology across locations. The *p*-values of multiple comparisons between groups at each location and between locations for each group were adjusted using Holm’s step-down Bonferroni method. A pairwise comparison of the differences between locations was reported for the VKC group only. *p* < 0.05 was considered statistically significant.

The associations between DCs and ocular surface symptoms and signs were initially assessed using univariate Spearman’s correlation (for the DC density and cell body size) and the Mann–Whitney U test (for the presence of dendrites). All five ocular surface locations were included in this initial analysis. Symptoms and signs that were significant at *p* < 0.01 (adjusted for multiple comparisons) were further analysed using a generalised estimating equation (GEE linear, ordinal, and binary logistic) model, that included location as an adjustment factor. No further multivariate modelling steps were carried out, due to the high multi-collinearity between symptoms, signs, and participant groups. Those symptoms and signs that were significantly associated with DC density and morphology at *p* < 0.05 in the GEE model are reported.

## 3. Results

Sixty participants completed the study, 20 each in the non-allergic (mean age: 26.7 ± 4.6 years, 70% male), AC (mean age: 27.1 ± 6.4 years, 75% male), and VKC (mean age: 13.4 ± 3.5 years, 65% male) groups. The VKC participants were significantly younger than the AC and non-allergic participants (*p* < 0.001). There was no significant difference in the distribution of gender between the groups (*p* = 0.80).

### 3.1. Clinical Findings

Table 1 summarises the symptoms and signs in the three groups. The total symptoms score (AUAQ) was highest in the VKC group, followed by the AC group (*p* = 0.01), and both were higher than in the non-allergic group (both *p* < 0.001). The VKC participants experienced worse burning, stinging, redness, and a need to rub their eyes than the AC participants (*p* ≤ 0.01). The participants in both the AC and VKC groups reported more symptoms of dryness, itchiness, burning, stinging, watering, redness, and a need to rub their eyes, compared to the non-allergic group (*p* ≤ 0.001). The participants in the VKC group had more limbal, bulbar, and palpebral redness compared to the AC group (*p* ≤ 0.01), and both were more severe than in the non-allergic group (*p* < 0.001). Conjunctival chemosis and follicles were higher in both the VKC and the AC group, compared to the non-allergic group (*p* ≤ 0.02), but conjunctival follicles were not significantly different between the VKC and AC groups. Corneal epithelial disorder and conjunctival papillae were observed only in VKC participants. 

### 3.2. DC Density

**Figure 1:**
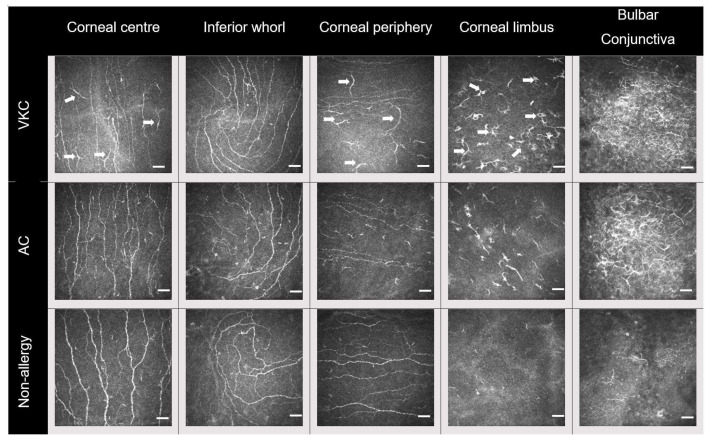
Representative in vivo confocal microscopy images of the corneal sub-basal and conjunctival epithelium in vernal keratoconjunctivitis (VKC, **top** row), allergic conjunctivitis (AC, **middle** row), and non-allergic (**bottom** row) participants. A higher dendritic cell (DC) density is evident in the AC and VKC groups compared to the non-allergic group, at all corneal and conjunctival locations. A higher DC density is observed at the corneal limbus in VKC compared to AC, as well as larger cell bodies in the corneal centre and on the periphery (arrows). In approximately half of the VKC and AC participants, the DCs at the bulbar conjunctiva tended to gather in wire netting clusters (**top right** and **middle right**). Image size = 400 × 400 µm; scale bar = 50 µm.

**Figure 2:**
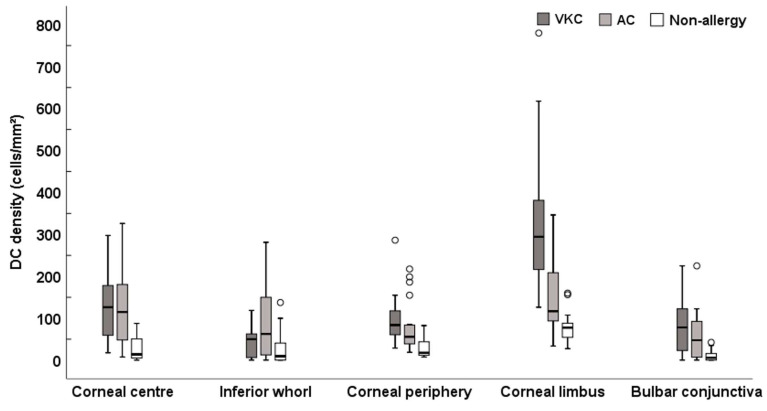
The dendritic cell (DC) density across corneal and conjunctival locations in 20 vernal keratoconjunctivitis (VKC), 20 allergic conjunctivitis (AC), and 20 non-allergic participants. The plots represent the median (horizontal black line), interquartile range (box), lower and upper extremes (whiskers), and outliers lying above Q3 + 1.5 * interquartile range and below Q1 − 1.5 * interquartile range (circles). * Indicates times.

Representative IVCM images at each location are shown in Figure 1. Linear mixed model analysis showed that the participant group had an impact on the DC density (F = 28.80, *p* < 0.001). A significant interaction between group and location (F = 3.60, *p* = 0.002) showed that the ocular surface location had an effect on the between-group difference in DC density. Simple main effects showed that both the AC and the VKC group had a higher DC density compared to the non-allergic group at all locations (*p* ≤ 0.01), except at the inferior whorl (*p* = 0.06). The DC density was numerically higher in the VKC group compared to the AC group at the corneal periphery, limbus, and conjunctiva, but this difference was significant only at the limbus (*p* < 0.001) (Figure 2 and Table 2).

In the VKC participants, there were significant differences in DC density between locations (F = 29.26, *p* < 0.001) (Table 2). The highest DC density was found at the limbus compared to all other locations (*p* ≤ 0.001). The inferior whorl had the lowest DC density compared to all other corneal locations (*p* ≤ 0.01), and it was numerically lower than the conjunctival DC density (*p* = 0.12). The conjunctival DC density was lower than the corneal limbus (*p* < 0.001), and it was numerically lower than the corneal centre (*p* = 0.16).

### 3.3. DC Morphology 

**Figure 3:**
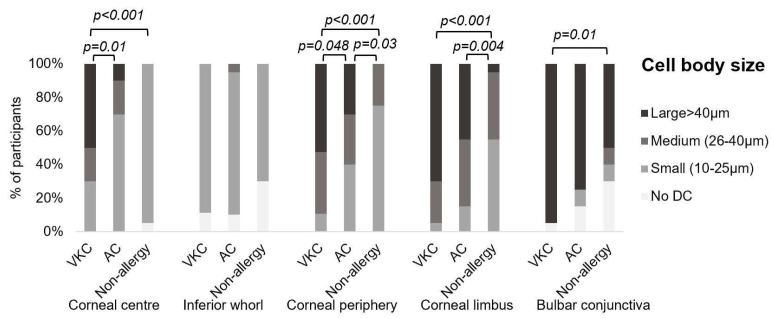
The dendritic cell (DC) body size across the vernal keratoconjunctivitis (VKC, *n* = 20), allergic conjunctivitis (AC, *n* = 20), and non-allergic (*n* = 20) groups. Differences between groups were apparent at the corneal centre, periphery, and limbus. Images devoid of DCs were not included in the DC morphology analysis.

The DC body size was larger in VKC and AC participants than in the non-allergic group. Significantly larger DC bodies were observed at the corneal periphery and limbus of the AC participants compared to the non-allergic group (*p* ≤ 0.03) and at the corneal centre, periphery, and limbus of the VKC participants compared to the non-allergic participants (*p* ≤ 0.001). Differences in DC body size between VKC and AC were apparent at the corneal centre and periphery, with larger cell bodies in the VKC participants (*p* < 0.05) (Figure 3).

There was a higher proportion of participants with DCs displaying dendrites and long dendrites in the cornea and conjunctiva in the VKC and the AC group compared to the non-allergic group. Significantly more participants with DCs exhibiting dendrites at the corneal centre and periphery were observed in both the VKC and AC groups compared to the non-allergic group (*p* ≤ 0.04). More participants with DCs displaying long dendrites at both the corneal periphery in AC (*p* = 0.01), and at the corneal centre, periphery, and limbus in VKC (*p* ≤ 0.001) were found compared to the non-allergic group. Participants with DCs exhibiting thick dendrites at the limbus were observed in greater numbers in the VKC and AC groups compared to in the non-allergic group (*p* ≥ 0.15) (Table 3). 

There were no significant differences between the VKC and AC groups in the frequency of participants with DCs presenting dendrites. Although the proportion of participants with corneal DCs displaying long dendrites and thick dendrites was frequently greater in the VKC group compared to the AC group, these differences were not shown to be statistically significant (*p* ≥ 0.08) (Table 3).

In 53% of VKC and 53% of AC participants, the DCs at the bulbar conjunctiva tended to gather in a “wire-netting pattern”; this feature was not evident in the non-allergic group. The majority of DCs in the bulbar conjunctiva had large cell bodies (Figure 3), and pairwise comparison showed that the VKC group had significantly larger cell bodies than the non-allergic group (*p* = 0.01). 

There were no significant differences in the proportion of participants with DCs presenting dendrites at the conjunctiva between groups (Table 3). In all three participant groups, almost all conjunctival DCs presented with dendrites, and the majority presented with long dendrites. Thick dendrites were not observed in the bulbar conjunctiva. 

In the VKC participants, significant differences in DC morphology were observed between locations (*p* ≤ 0.001). At the inferior whorl, the DC bodies were significantly smaller than those at other locations (*p* ≤ 0.01). The DC bodies tended to be larger at the conjunctiva of VKC participants, followed by at the corneal limbus, periphery, and corneal centre, although the differences in cell body size between these locations were not significant. DCs with dendrites were observed in all VKC participants at all locations, except at the inferior whorl (31% of VKC participants). DCs with long dendrites were present in more than half of the VKC participants at the corneal centre, and in more than 80% of the VKC participants at the corneal periphery, limbus, and conjunctiva. Thick dendrites were observed at the corneal limbus in half of the VKC participants.

### 3.4. Associations between DC Density and Morphology and Ocular Surface Symptoms and Signs

Almost all symptoms and signs showed a moderate correlation with DC density and DC body size (rho ≤ 0.40, *p* ≤ 0.003), the presence of dendrites, and the presence of long dendrites (*p* ≤ 0.001), and only corneal epithelial disorders and bulbar conjunctival chemosis were correlated with the presence of thick dendrites (*p* ≤ 0.009) at the univariate level (Supplementary Table S1). Also, univariate modelling showed a significant correlation between the symptoms and signs and DC density and morphology that were significant at the univariate level (the cell body size, presence of dendrites, and presence of long dendrites) (*p* ≤ 0.02).

## 4. Discussion

In this study, the DC density and morphology were assessed and compared across various types of ocular allergy. The DC density was higher in the corneal limbus of VKC participants compared to those with AC, and both the VKC and the AC participants had a higher DC density at corneal and conjunctival locations compared to the non-allergic group. The DC morphology was also observed to be somewhat pathognomonic, with larger cell bodies observed at the corneal centre and periphery in those with VKC compared to those with AC. Compared to the non-allergic group, larger cell bodies and a higher proportion of participants with DCs presenting dendrites and long dendrites were evident at the corneal locations in both the VKC and AC groups.

In both the VKC and AC groups, there was a higher DC density compared to the non-allergic group at all corneal and conjunctival locations except the inferior whorl. The VKC group had a significantly higher DC density compared to the AC group only at the corneal limbus. In line with the current findings, a higher DC density at the central, peripheral cornea, limbus, tarsal and bulbar conjunctiva in VKC participants compared to a normal group has been previously reported [12,13,14]. We have recently shown a higher DC density at the cornea and bulbar conjunctiva in participants with AC [11]. This is the first time that VKC and AC have been directly compared.

In VKC patients, the DC morphology was briefly described by other IVCM studies as comprising highly reflective cells with dendritic processes [13,14]. This study provides one of the first comprehensive analyses of DC morphology in ocular allergy conducted with IVCM. An altered DC morphology was observed, with larger cell bodies and a higher proportion of participants with DCs exhibiting dendrites and long dendrites at the corneal locations in the VKC and AC groups compared to in the non-allergic group, likely representing an increased capacity for antigen capture [19]. Very few differences in DC morphology could be observed between VKC and AC, other than that the DC bodies at the corneal centre and periphery in VKC appeared larger than in AC. A higher proportion of participants with DCs displaying long dendrites was also observed in the VKC group compared to AC; however, the study did not have the power to detect these differences. We have previously reported larger DC bodies and a higher proportion of participants with DCs exhibiting long dendrites at the corneal locations in AC compared to in non-allergic controls [11]. Longer dendrites in the corneal centre of a group of participants with a systemic allergy, characterised by a positive skin prick test, compared to non-allergic participants, have also been reported [17]. The wire-netting pattern of DCs at the conjunctiva of both VKC and AC may represent a pattern specific to inflammatory response at the conjunctiva, as it was not observed in the non-allergic group. A cluster of DCs at the bulbar conjunctiva of VKC patients has been previously reported [13].

DCs are essential to the initiation of the allergic response [7,8]. Activated CD4+ T cells, DCs, macrophages (CD68+), and an increased HLA-DR expression have been reported in VKC [4]. Different types of conjunctival DCs in a mouse model of AC, following an allergen challenge, have been demonstrated [6,10]. The current study confirmed DC activation in both VKC and AC. Understanding the dissimilarity in the immunopathologic mechanisms of different forms of ocular allergy may be important to increasing our understanding of disease, as it may help to improve the management of allergic eye diseases.

A majority of DCs at the corneal periphery and limbus of the VKC group displayed medium-to-large cell bodies. All DCs at the corneal centre, periphery, and limbus had dendrites, and a majority had long dendrites. Thick dendrites were also observed in half of the VKC participants. In total, 95% of VKC participants had conjunctival DCs, and almost all had large cell bodies. These conjunctival DCs had dendrites, and a majority of them were long. Long dendrites have a higher antigen capture capacity, and thick dendrites show a higher migratory capacity [19,26,27,28]. Thus, in the current study, it can be concluded that DCs at the bulbar conjunctiva, limbus, and periphery, as well as the corneal centre, in VKC participants, are likely to show a high capacity for antigen capture, while those at the corneal limbus are likely to show a high migratory capacity.

Moderate associations between DCs and ocular surface symptoms and signs of allergy were observed in the current study. In previous studies involving systemic-allergic and AC participants, no such correlation was found [11,17]. These differing results may be explained by the more severe allergy manifestations in the participants enrolled in the current study. The moderate association found between DCs and ocular surface symptoms and signs of allergy suggests that the consideration of DCs as a biomarker of ocular allergic disease may be warranted; however, further evaluation is needed to elucidate which of the DC characteristics described in this study (density, morphology) is most relevant.

A higher DC density and altered morphology in the AC group compared to the non-allergic group aligns with our previous findings in AC in Australia [11]. However, the AC group in the current study (Iran) had a higher DC density at the corneal and conjunctival locations and slightly differing DC morphological characteristics at the corneal limbus, with larger DC bodies and a higher proportion of participants with DCs exhibiting thick dendrites compared to the corresponding Australian group in the previous study [11]. Several factors may account for these differences. In the current study, recruitment was clinic-based; those who attended an eye clinic and were clinically diagnosed with AC by their treating ophthalmologist were opportunistically recruited and, thus, they may represent a more severe form of AC. In contrast, those participants with AC enrolled in the Australian study were recruited from the general community. In support of this, a cursory examination and comparison of signs and symptoms revealed that the Iran cohort tended to display more severe symptoms (AUAQ total symptom scores, Iran group: median (range); 10 (0–18), Australia group 5 (0–10)) and a higher bulbar redness (Iran group: median (range); 2.9 (1.8–3.7), Australia group 2.4 (1.7–3.4) than the AC participants from the Australian study. The AC participants in the current study were of Middle Eastern ethnicity, whereas in the Australian study, the participants were of multiple ethnicities (Australia, New Zealand, North America, Central America, East Asia, Southeast Asia, and South Asia). These population differences suggest that the immune response could be modulated by disease severity, genetics/ethnicity, or environmental factors. A similar population difference in DC density was suggested in an earlier study [21].

The data collection for the current study occurred during the COVID-19 pandemic in Iran. Participants were screened for the COVID infection, and only those who had not been infected a month before the study visit were included. Nevertheless, a previous episode of either symptomatic or asymptomatic COVID-19 infection could have impacted the DC density and morphology. Ocular manifestations are commonly reported by COVID-19 patients [29]. An increased central DC density has been reported in COVID-19 patients, and persisted at 12 weeks in those with chronic neurological symptoms [30]. The use of face masks was ubiquitous amongst participants of the current study. Whilst the impact of mask-wearing on DCs remains unknown, studies have reported that face mask usage can dampen nasal and ocular allergy symptoms in allergic rhinitis patients [31,32].

The VKC participants were younger than the participants in the AC and non-allergic groups. Age could have impacted the DC density and morphology; however, this is unlikely, given that previous evidence showed that age did not have an effect on the central DC density [21]. Longitudinal studies examining the effect of VKC on DC density and morphology over time are required in order to investigate this further.

The AC participants were included based on their medical history and clinical signs or a previous diagnosis of AC, without confirmation of their allergy status using diagnostic tests such as a skin prick test. The VKC participants were also included based on their clinical signs and history, and the presence of Horner–Trantas dots. The VKC group was thus limited to the limbal form of the disease. The generalisability of these findings to other forms of VKC is unclear.

Even though IVCM has the distinct advantage of enabling the visualisation of living tissue, it lacks the ability to verify cell phenotypes and provide essential information about cell surface markers, to confirm the identity of the observed cells as DCs. In a recently published study, some DCs lacking dendrites, observed in the corneas of both post mortem human subjects and mice, were identified as resident memory T cells [33]. The presumed generalisability of these findings to the live human cornea requires additional investigation. While the verification of these results through other methods, such as immunohistochemistry, is unfeasible for living human corneal tissue, it remains a viable option for conjunctiva.

In summary, the current study demonstrated a potentially important pathognomonic role of corneal and conjunctival DCs in both VKC and AC. Based on these findings, it is proposed that DC density and morphology can be used to differentiate VKC from AC. Moreover, this study provided strong foundations for further studies into ocular allergy (the time course, wide spectrum of disease), and to explore the usefulness of DC density, morphology, and topographical distribution as markers of the efficacy of allergy treatments.

## Figures and Tables

**Table 1:** Summary of the findings for ocular allergy symptoms and signs in 20 vernal keratoconjunctivitis (VKC), 20 allergic conjunctivitis (AC), and 20 non-allergic participants. The values are reported as the median (range). The ocular signs were graded in integers except for limbal, bulbar, and palpebral redness, which were graded in 0.1 steps. Statistically significant values (*p* < 0.05) are indicated in bold/italics. AUAQ, Aston University Allergy Questionnaire. † ‡ # indicates statistically significant pairwise comparisons.

		VKC (*n* = 20)	AC (*n* = 20)	Non-Allergy (*n* = 20)	*p*-Value
Ocular surface symptoms	AUAQ, Total symptom score (0–21)	16 (13–19) ‡#	10 (0–18) †#	1 (0–3) †‡	***p < 0.001 ***
	Dryness (0–3)	1 (0–2) ‡	1 (0–2) †	0 (0–1) †‡	***p < 0.001 ***
	Itchiness (0–3)	3 (2–3) ‡	2 (0–3) †	0 (0–0) †‡	***p < 0.001 ***
	Burning (0–3)	3 (2–3) ‡#	1 (1–3) †#	0 (0–1) †‡	***p < 0.001 ***
	Stinging (0–3)	2 (1–3) ‡#	1 (0–3) †#	0 (0–0) †‡	***p < 0.001 ***
	Watering (0–3)	1 (1–3) ‡	1 (0–2) †	0 (0–1) †‡	***p < 0.001 ***
	Redness (0–3)	3 (2–3) ‡#	2 (0–3) †#	0 (0–1) †‡	***p < 0.001 ***
	A need to rub eyes (0–3)	3 (2–3) ‡#	1 (0–3) †#	0 (0–0) †‡	***p < 0.001 ***
Ocular surface signs	Limbal redness (0–4, 0.1)	4.0 (3.0–4.0) ‡#	2.9 (1.5–4.0) †#	1.2 (0.5–2.0) †‡	***p < 0.001 ***
	Bulbar redness (0–4, 0.1)	3.7 (2.8–4.0) ‡#	2.9 (1.8–3.7) †#	1.3 (0.7–2.0) †‡	***p < 0.001 ***
	Palpebral redness (0–4, 0.1)	3.5 (3.0–4.0) ‡#	2.6 (1.5–4.0) †#	1.2 (0.5–2.0) †‡	***p < 0.001 ***
	Corneal epithelial disorder (0–3, 1)	1 (0–2) ‡#	0 (0–0) #	0 (0–0) ‡	***p < 0.001 ***
	Bulbar conjunctival chemosis (0–3, 1)	1 (1–2) ‡#	1 (0–2) †#	0 (0–1) †‡	***p < 0.001 ***
	Palpebral conjunctival papillae (0–3, 1)	2 (1–2) ‡#	0 (0–0) #	0 (0–0) ‡	***p < 0.001 ***
	Palpebral conjunctival follicle (0–3, 1)	0 (0–2) ‡	0 (0–2) †	0 (0–0) †‡	***p = 0.01 ***

**Table 2:** The dendritic cell density at corneal and conjunctival locations in 20 vernal keratoconjunctivitis (VKC), 20 allergic conjunctivitis (AC), and 20 non-allergic participants. The data are presented as the median (IQR). Statistically significant values (*p* < 0.05) are indicated in bold/italics.

Location	Dendritic Cell Density (Cells/mm^2^)			Pairwise Comparison		
	VKC (*n* = 20)	AC (*n* = 20)	Non-Allergy (*n* = 20)	Non-Allergy vs. AC	Non-Allergy vs. VKC	AC vs. VKC
Corneal centre	126.2 (57.2–187.2)	115.0 (37.2–181.6)	13.7 (5.0–51.0)	***0.001 ***	***0.001 ***	0.70
Inferior whorl	50.0 (6.2–67.2)	62.5 (12.5–153.1)	9.4 (0–42.2)	0.06		
Corneal periphery	83.7 (56.2–122.5)	55.6 (38.7–84.4)	17.5 (11.6–44.4)	***0.001 ***	***0.001 ***	0.20
Corneal limbus	294.4 (210.0–383.7)	116.9 (86.9–210.0)	77.5 (53.4–88.4)	***0.002 ***	***0.001 ***	***0.001***
Bulbar conjunctiva	78.1 (21.6–122.5)	47.5 (6.6–92.5)	5.6 (0–16.0)	***0.01 ***	***0.001***	0.15

**Table 3:** The corneal and conjunctival dendritic cell (DC) morphology for the presence of dendrites, and the presence of long and thick dendrites in the vernal keratoconjunctivitis (VKC, *n* = 20), allergic conjunctivitis (AC, *n* = 20), and non-allergic (*n* = 20) groups. Images devoid of DCs were not included in the DC morphology analysis and, thus, data are presented for participants with DCs at each location. Statistically significant values (*p* < 0.05) are indicated in bold/italics. NA indicates not applicable.

	% of Participants			Pairwise Comparisons		
	VKC *n* = 20	AC *n* = 20	Non-Allergy *n* = 20	Non-Allergy vs. AC	Non-Allergy vs. VKC	AC vs. VKC
**Presence of dendrites**						
Corneal centre	100%	85%	42%	***0.02***	***0.001***	0.30
Inferior whorl	31%	33%	7%	0.31	0.35	1.0
Corneal periphery	100%	95%	60%	**0.04**	***0.01***	1.0
Corneal limbus	100%	100%	100%	NA	NA	NA
Bulbar conjunctiva	100%	100%	86%	0.40	0.40	NA
** Presence of long dendrites **						
Corneal centre	60%	35%	5%	0.09	***0.001***	0.20
Inferior whorl	6%	5%	0	1.0	1.0	1.0
Corneal periphery	84%	55%	10%	***0.01***	***0.001***	0.08
Corneal limbus	90%	70%	30%	0.052	***0.001***	0.25
Bulbar conjunctiva	84%	88%	50%	0.13	0.13	1.0
** Presence of thick dendrites **						
Corneal centre	0	0	0	NA	NA	NA
Inferior whorl	0	0	0	NA	NA	NA
Corneal periphery	10%	0	0	NA	0.50	0.50
Corneal limbus	55%	40%	20%	0.60	0.15	0.60
Bulbar conjunctiva	5%	0	0	NA	1.0	1.0

## Data Availability

Data are available upon reasonable request.

## Supplementary Materials

The following supporting information can be downloaded at: https://www.mdpi.com/article/10.3390/biom13101469/s1, Table S1: The association between dendritic cell density/morphology and ocular surface symptoms/signs examined using Spearman correlation and the Mann–Whitney U test as appropriate in 60 VKC, AC, and non-allergic participants. All five locations were included; therefore, the level of significance was set at *p* < 0.01 (adjusted for multiple comparisons).
