# Supplementary material for: Dendritic Cell Density and Morphology Can Be Used to Differentiate Vernal Keratoconjunctivitis from Allergic Conjunctivitis
Source: Biomolecules. 2023 Sep 29;13(10):1469. doi: 10.3390/biom13101469 (PMC10605082; doi:10.3390/biom13101469)
Supplement: Supplementary file 1 [file biomolecules-13-01469-s001.zip › biomolecules-2603219-supplementary.pdf]

Supplementary TableS1: Association between dendritic cell density/ morphology and ocular surface symptoms/ signs examined using Spearman correlation and Mann-Whitney U test as appropriate in 60 participants with VKC, AC, and non-allergic. All 5 locations were included, therefore the level of significance was set at p<0.01 (adjusted for multiple comparisons).

	Density	DC morphology			
		Cell body size	Presence of dendrites	Presence of long dendrites	Presence of thick dendrites
Dryness	***rho=0.17 p=0.003***	***rho=0.19 p=0.001***	p=0.17	***p=0.001***	p=0.25
Itchiness	***rho=0.35 p=0.001***	***rho=0.33 p=0.001***	***p=0.001***	***p=0.001***	p=0.05
Burning	***rho=0.33 p=0.001***	***rho=0.32 p=0.001***	***p=0.001***	***p=0.001***	p=0.03
Stinging	***rho=0.40 p=0.001***	***rho=0.38 p=0.001***	***p=0.001***	***p=0.001***	p=0.012
Watering	***rho=0.35 p=0.001***	***rho=0.28 p=0.001***	***p=0.001***	***p=0.001***	p=0.02
Redness	***rho=0.38 p=0.001***	***rho=0.36 p=0.001***	***p=0.001***	***p=0.001***	p=0.013
A need to rub eyes	***rho=0.39 p=0.001***	***rho=0.36 p=0.001***	***p=0.001***	***p=0.001***	p=0.03
AUAQ, Total symptom score	***rho=0.37 p=0.001***	***rho=0.34 p=0.001***	***p=0.001***	***p=0.001***	p=0.012
Limbal redness	***rho=0.39 p=0.001***	***rho=0.38 p=0.001***	***p=0.001***	***p=0.001***	p=0.02
Bulbar redness	***rho=0.40 p=0.001***	***rho=0.38 p=0.001***	***p=0.001***	***p=0.001***	p=0.02
Palpebral redness	***rho=0.38 p=0.001***	***rho=0.37 p=0.001***	***p=0.001***	***p=0.001***	p=0.02
Corneal epithelial disorders	***rho=0.31 p=0.001***	***rho=0.31 p=0.001***	***p=0.002***	***p=0.001***	***p=0.009***
Bulbar conjunctival chemosis	***rho=0.34 p=0.001***	***rho=0.34 p=0.001***	***p=0.001***	***p=0.001***	***p=0.002***
Palpebral conjunctival papillae	***rho=0.30 p=0.001***	***rho=0.32 p=0.001***	***p=0.003***	***p=0.001***	p=0.03
Palpebral conjunctival follicle	***rho=0.23 p=0.001***	***rho=0.18 p=0.003***	p=0.04	***p=0.001***	p=0.85
